# Qualitative and Quantitative Evaluation of Dietary Intake in Patients with Non-Alcoholic Steatohepatitis

**DOI:** 10.3390/nu9101074

**Published:** 2017-09-28

**Authors:** Alessandro Federico, Marcello Dallio, Giuseppe Gerardo Caprio, Antonietta Gerarda Gravina, Desiree Picascia, Mario Masarone, Marcello Persico, Carmela Loguercio

**Affiliations:** 1 Department of Clinical and Experimental Medicine, University of Campania “L. Vanvitelli”, Via Pansini 5 80131 Naples, Italy; marcello.dallio@gmail.com (M.D.); giuseppegerardo.caprio@hotmail.it (G.G.C.); antonietta.gravina@yahoo.it (A.G.G.); dpicascia90@gmail.com (D.P.); carmelina.loguercio@unicampania.it (C.L.); 2 Department of Medicine and Surgery, University of Salerno, Via Salvador Allende 84081 Baronissi, Salerno, Italy; mmasarone@unisa.it (M.M.); mpersico@unisa.it (M.P.)

**Keywords:** non-alcoholic steatohepatitis, dietary intake, micronutrients, macronutrients

## Abstract

There are very few reports about the intake of nutrients for the development or progression of non-alcoholic steatohepatitis (NASH). The aim of this study was to identify the dietary habits and the nutrient intake in patients with NASH, in comparison to chronic hepatitis C (HCV)-related patients. We prospectively evaluated the intake of macronutrients and micronutrients in 124 NAFLD and 162 HCV patients, compared to 2326 subjects as a control group. We noticed major differences in macro- and micronutrients intakes in NASH and HCV patients compared to controls. Proteins, carbohydrate (glucose, fructose, sucrose, maltose and amide), saturated fatty acid (SFA), monounsaturated fatty acid (MUFA), folic acid, vitamin A and C (*p* < 0.0001), and thiamine (*p* < 0.0003) ingestion was found to be higher in patients with NASH, while total lipids, polyunsaturated fatty acid (PUFA), riboflavin and vitamin B6 daily intake were lower compared to controls (*p* < 0.0001). Similarly, NASH patients had significantly reduced carbohydrate intake (*p* < 0.0001) and an increased intake of calcium (*p* < 0.0001) compared to HCV positive patients. Finally, we showed in NASH males an increase in the intake of SFA, PUFA, soluble carbohydrates (*p* < 0.0001) and a decrease in the amount of fiber (*p* < 0.0001) compared to control males. In NASH female population, we showed an increase of daily total calories, SFA, MUFA, soluble carbohydrates, starch and vitamin D ingested (*p* < 0.0001) with a reduction of fibers and calcium (*p* < 0.0001) compared to control females. This study showed how NASH patients’ diets, in both male and females, is affected by a profound alteration in macro- and micronutrients intake.

## 1. Introduction

Non-alcoholic fatty liver disease (NAFLD) is defined as a progressive disease caused by an increased fat storage in the liver of patients who do not consume excessive alcohol and do not have any type of virus related liver disease [1]. It is considered the hepatic manifestation of metabolic syndrome [2], and represents a growing challenge in terms of prevention and treatment. The prevalence of NAFLD is rapidly increasing, ranging 20–30% in the general population [3]; it is strongly associated with obesity and is found in up to 91% of severely obese patients undergoing bariatric surgery, and up to 5% of these patients may have unsuspected cirrhosis [4]. This is also an emerging problem in pediatric population: in a recent review, Nobili et al. highlighted an approximate three-fold increase in childhood obesity, with a dramatic rise in NAFLD from 5.0% in 1960 to 15.4% in 2001 and 16.9% in 2009/2010 [5]. Depending on various factors such as physical activity and particularly ethnic origin, even lean subjects may have visceral obesity in significant proportions [6], with potential risk for insulin resistance and other metabolic syndrome characteristics [7], and non-alcoholic steatohepatitis (NASH) affects almost half of the severely obese population. Its close association not only with obesity but also with insulin resistance and dyslipidemia led to the hypothesis that NAFLD is the hepatic manifestation of metabolic syndrome [8].

Although the mechanisms involved in the pathogenesis of NAFLD are still not fully understood, the most important theory explaining the physiopathology of NAFLD is the “multiple hit” hypothesis that considers multiple strikes/hits acting together on genetically predisposed subjects such as insulin resistance, hormones secreted from the adipose tissue, gut microbiota, and especially nutritional factors [9,10,11,12].

The individual roles of various nutritional and metabolic factors in the pathogenesis and natural history of NAFLD are still incompletely understood [13,14,15,16,17]. Diet influences body mass index (BMI), iron content in the liver, insulin, enzyme activities, substrate reserves, and metabolic pathways in hepatocytes, and many nutrients have been reported to exert protective or toxic effects on the liver in animal models and humans [18,19,20,21,22].

Some dietary components can directly activate inflammatory pathways. An increase in the amount of fat introduced by a daily diet is related to an increase in the development of metabolic liver disease, not only in relation to their liver accumulation, but also acting as stimulus to trigger inflammation and thus liver dysfunction. Free fatty acids (FFA) induce lysosomal instability, leading to release of cathepsin B and nuclear factor (NF)-κB activation. Palmitic acid activates interleukin (IL)1β and IL18 through a pathway involving toll like receptor (TLR)2 and cryopyrin (NALP3) inflammasome. The aryl hydrocarbon receptor (AhR) provides an important link between the intestinal immune system and food-derived ligands. AhR activating ligands include indolo[3,2-b] carbazole or 6-formylindolo[3,2-b]carbazole, which comes from cruciferous vegetables; flavonoids; polyphenols; bacterial-derived molecules, such as phenazins and naphtoquinon phthiocol; and microbial metabolic products, such as 1,4-dihydroxy-2-naphthoic acid. AhR-induced production of IL22 could have immunomodulatory, anti-inflammatory and metabolic effects [23].

Patients with uncomplicated steatosis and NASH have been studied to discern common dietary patterns and macronutrient intake. There are several tools available, including diet records, weighed diet records, 24-h dietary recall, food frequency questionnaires, and combined methods to assess nutrients intake [24]. The food frequency questionnaire is the most used and efficient method to evaluate relations between food intake and diseases [25].

Individuals with uncomplicated steatosis and NASH have been studied to discern common dietary patterns and macronutrient intake. While some studies revealed elevated carbohydrate and fat intake, scientific and clinical consensus is lacking. Dietary assessment cannot reveal lipotoxicity per se [18], but it is strongly related to the development of obesity and insulin resistance [22,26]. It has been reported that diets with a high glycemic index and glycemic load are positively associated with insulin resistance and intake of simple carbohydrates is also associated with insulin resistance [27].

Guidelines of the European Association of Liver Diseases (EASL) highlight the importance of diet in the pathogenetical mechanism leading to NAFLD occurrence: in fact, EASL guidelines recommend weight loss of at least 7–10% in overweight/obese NAFLD and exclusion of NAFLD-promoting components, such as processed food, or beverages high in added fructose, emphasizing the adherence to the so-called “Mediterranean diet” [28].

However, there are few reports about the intake of several nutrients for the development or progression of NASH. Moreover, unbalanced diets were found to affect the development and progression of disease in a group of patients with NASH [29]. Other studies have shown that both quantity and quality of dietary fat influences liver fatty acid synthesis [30,31,32] and insulin sensitivity [33,34]. Dietary short chain fatty acids can induce insulin resistance [35] and raise the risk of cardiovascular disease [2]. The accumulation of short fatty acids as triglycerides in non-adipocyte cells leads to cellular damage as a result of their lipotoxicity. Triglyceride accumulation in skeletal muscle cells for example is also associated with deficient fatty acids oxidation. The biochemical mechanisms responsible for lower fatty acid oxidation involve reduced carnitine palmitoyl transferase (CPT) activity, as a likely consequence of increased intracellular concentrations of malonyl-CoA, reduced glycogen synthase activity and is associated to impairment of insulin signalling and glucose transport. However, a threshold above which this effect is seen has yet to be determined [35].

The aim of this study was to identify the dietary habits and the nutrient intake in patients with NAFLD, in comparison to chronic hepatitis C (HCV)-related patients and a control healthy group to produce a picture of real life dietary habits in a large population study. Moreover, we evaluated the differences in this assessment between male and female patients to identify the presence or not of statistically significant differences related to the pathogenesis of liver diseases. The results of our study could be useful to understand the role and the support that a particular eating habits could have in the development and progression of different types of chronic liver disease.

## 2. Materials and Methods

We enrolled 124 patients affected by NAFLD and 162 HCV from Southern Italy referred to our Department between January 2014 and December 2015. Patients with diabetes as well as patients with severe renal, hepatic or cardiovascular disease were excluded from the study. Subjects taking hypolipidemic, immunosuppressive or glucose lowering drugs as well as patients with a history of excessive drinking or other liver diseases, including alcoholic liver disease, viral hepatitis, autoimmune hepatitis, primary biliary cirrhosis, hemochromatosis, Wilson’s disease, or α1-antitrypsin deficiency were excluded from the study.

With reference to HCV patient, we excluded from the study patients with decompensated cirrhosis, and patients with such other clinically relevant associated diseases as decompensated diabetes, kidney diseases, pulmonary diseases, collagen diseases and cancer. HCV genotypes were collected before starting antiviral treatment. In the control group, we studied 2326 apparently healthy blood donors who were negative for HCV antibody and hepatitis B surface antigen (HBsAg), and had normal liver tests. In NAFLD/HCV patients liver biopsies were performed about 6 months before enrolment.

The study protocol was approved by the local ethics committee and all patients gave their informed written consent before participation. The ethic approval code is 2005-000860-24.

### 2.1. Clinical and Laboratory Measurements

Blood samples were drawn after an overnight fast, rapidly processed or stored at −20 °C after centrifuging. Glucose, insulin, aspartate aminotransferase (AST) and alanine aminotransferase (ALT) levels were measured enzymatically using commercially available kits (R&D Systems, Minneapolis, MN, USA). Insulin sensitivity was estimated by Homeostatic model assessment (HOMA) index which was calculated using the formula:

HOMA = fasting insulin (μU/mL) × plasma glucose (mmol/L)/22.5 [36].

### 2.2. Body Mass Index (BMI)

BMI was calculated as body weight (kilograms) divided by body height (meters) squared. Patients and controls with a BMI between 25 kg/m^2^ and 29.9 kg/m^2^ were considered overweight, and those with a BMI ≥ 30 kg/m^2^ were considered obese.

### 2.3. Nutrient Intake

Dietary habits were recorded by all enrolled subjects. There are several tools available, including diet records, weighed diet records, 24 h dietary recall, food frequency questionnaires, and combined methods to assess intake of nutrients [34,35]. Among these methods, the food frequency questionnaire is used most often to evaluate relations the relationship between food intake and diseases. The food intake of a complete week, including working days and the weekend, was recorded by each subject using a weekly dietary diary. In detail, we requested in each diary questionnaire the precise amount of portions size in terms of grams and the cooking methods. Moreover, we asked participants to take a photos of the nutritional values of each product utilized consumed and all participants were shown, at the start of the study, how to complete the diary accurately during the assessment and we showed, before the beginning of the study, the correct modality of how to record dietary information in the diary. These were converted to nutrient and food intake data using a computer program. For this purpose, we used the Winfood Software 2.0 package (Medimatica s.r.l., Martinsicuro, Italy), which has previously been used to assess alimentary dietary habits [37,38]. Because of the quantities and qualities of foods consumed, the program estimates the energy intake and the percentage of macronutrients and micronutrients, and calculates the elements nutrients in each food. Proteins are reported as animal and vegetable proteins. Carbohydrates are divided into soluble and amide. Lipids are divided into saturated, polyunsaturated, monounsaturated fatty acids and cholesterol. The complete elaboration estimation of intakes shows the list of diet components, the ratio between components and calories, and the subdivision of breakfast, lunch and dinner. The data were compared with the tables of food consumption and recommended dietary intakes of the Italian National Institute of Nutrition and Food Composition Database in Italy [39].

### 2.4. Histological Assessment

Absence or presence of NASH was evaluated according to standard histopathologic criteria and severity of the disease was assessed using the NAS (non-alcoholic fatty liver disease activity score) established by Kleiner [40], as the sum of scores of steatosis, lobular inflammation and hepatocellular ballooning. NASH was considered as diagnostic for NAFLD activity score (NAS) ≥ 5. The 152 patients with HCV-correlated chronic liver disease underwent a liver biopsy which was scored according to Ishak et al [41]. Fibrosis was scored according to Brunt et al. [42], as stage 0 (none), stage 1 (zone 3 perisinusoidal or portal fibrosis), stage 2 (zone 3 perisinusoidal and periportal fibrosis without bridging), or stage 3 (bridging fibrosis). Hepatocyte ballooning was scored as 0 (none), 1 (few) or 2 (many).

## 3. Statistical Analysis

Descriptive data are expressed as mean ± Standard Deviation. Kolgoromov–Smirnov Goodness-of-Fit (K-S) test was performed to evaluate normal distribution of the variables. Two-independent-samples *t*-test comparison of means, unequal variances assumed, with Welch approximation was used to evaluate the difference between groups: NAFLD and HCV group; NAFLD and controls; and HCV and controls. For all calculations, statistical significance was inferred at a variable two tailed *p*-value (*p* < 0.0001) (with a 95% of confidence interval), depending on the number of variables included in each table on the basis of a Bonferroni multiple comparison post-hoc correction (α/n), except for the Table 1, where we defined a *p*-value of 0.05. Moreover, we performed the evaluation of the power of our sample. In a two-way analysis of two paired samples with an alpha 0.05 with a 95% confidence interval (CI), the 1-Beta of our analyses was far above 0.9: in particular, it was 1 in controls vs. both NAFLD and HCV; 0.94 in male vs. females in NAFLD; and 0.93 in males vs. females HCV. All calculations were performed using Stata Statistical Software: Release 10© for Macintosh^®^ (StataCorp LP, College Station, TX, USA).

## 4. Results

Table 1 shows the baseline demographic and metabolic data of NASH, HCV patients and controls (age was expressed as median because this parameter was not normally distributed). The BMI is different among the groups. There are no differences in percent overweight but 15% of males and 18% of females in the NASH group are obese, as well as 12% of male and 6% of women in HCV group. The HOMA index, as for AST, ALT and GGT levels, are higher in NASH subject, compared to control groups. The differences of HOMA index in our population study were found to be statistically significant after correcting the HOMA and liver function data for BMI.

Table 2 and Table 3 show the intake of macronutrients and micronutrients in patients and controls together with the daily amounts recommended in Italy. We noticed major differences in macro- and micronutrients intakes in NASH and HCV patients compared to controls. The daily amount of total calories was not significantly different among groups, but proteins, carbohydrates (glucose, fructose, sucrose, maltose and amide), saturated fatty acid (SFA), monounsaturated fatty acid (MUFA), folic acid, vitamin A and C (*p* < 0.0001) and thiamine (*p* < 0.0003) ingestion was found to be higher in patients with metabolic liver disease, while total lipids, polyunsaturated fatty acid (PUFA), riboflavin and vitamin B6 daily intake were lower compared to controls (*p* < 0.0001); similar results emerged in HCV positive patients, with the addition of a reduced carbohydrate (glucose, fructose, sucrose and maltose) intake (*p* < 0.0001) and an increased intake of calcium (*p* < 0.0001).

Table 4 and Table 5 show the intake of macro- and micronutrients in NASH patients and HCV-positive patients compared to control, after splitting them according to their gender; even in this case, we found some significant differences about the patients alimentary choices, similar to the ones that emerged in previous tables. Female NASH patients showed a statistically significant increase of daily total calories, saturated fatty acids, MUFA, total carbohydrates, and amide and soluble carbohydrates (glucose, fructose, sucrose, and maltose); and a reduction in total proteins and animal proteins, total lipids, PUFA, and fiber in comparison to controls. Male NASH patients showed a statistically significant increase of daily saturated fatty acids, MUFA, and total carbohydrates; and a reduction of total proteins, total lipids, PUFA, soluble carbohydrates (glucose, fructose, sucrose, and maltose) and fiber in comparison to controls.

Female HCV patients showed a statistically significant increase of daily animal proteins, saturated fatty acids, and MUFA; and a reduction of total proteins and vegetable proteins, total lipids, PUFA, total carbohydrates and fiber in comparison to controls. Male HCV patients showed a statistically significant increase of daily saturated fatty acids and MUFA; and a reduction of total proteins, total lipids, PUFA, total carbohydrates and soluble carbohydrates (glucose, fructose, sucrose, and maltose) in comparison to controls. Moreover, female NASH patients showed a statistically significant increase of folic acid, thiamine, vitamin D and a reduction of zinc, riboflavin, vitamin B6 in comparison to controls. Male NASH patients showed a statistically significant increase of Vitamin A and C in comparison to controls. Female HCV patients showed a statistically significant increase of folic acid, thiamine, and vitamin B6; and a reduction of zinc and vitamin D in comparison to controls.

Male HCV patients showed a statistically significant increase of vitamin A and a reduction of Vitamin B6 and C in comparison to controls.

Finally, to better assess the differences between the two liver disorders, in Table 6 and Table 7, we compared NASH and HCV positive patients divided by gender. We also noticed in this case that substantial differences about the dietary habits in both categories were found. The most significant differences were found in NASH males, concerning a higher intake of SFA, PUFA, MUFA, and soluble carbohydrates (*p* < 0.0001); and a reduced intake of fiber (*p* < 0.0001). In addition, an increased intake of total calories, SFA, MUFA, soluble carbohydrates, starch and vitamin D (*p* < 0.0001), with a significantly reduced amount of fiber and calcium in NASH (*p* < 0.0001), compared to HCV males, was found.

## 5. Discussion

Dietary components have been implicated in affecting liver functioning both in physiological conditions [43] and in patients with liver diseases [44,45,46,47]. The aim of our research, conducted on NASH and HCV related chronic hepatitis patients, was to further explore dietary habits of subject affected by liver disease and how changes in recommended nutrient intake could be the basis for the occurrence of these pathologies.

In fact, changes in carbohydrate, lipid, protein, vitamin and oligo-element intake have been widely discussed as fundamental factors for developing and maintaining metabolic hepatopathy.

In relation to macronutrients, our study highlights that NASH patients eat more proteins and more carbohydrates (glucose, fructose, sucrose, maltose and amide) than controls: with reference to the first one, various studies showed that an enriched protein diet could improve glucose homeostasis and help weight loss [48]; however, it is important to consider the animal to vegetable protein ratio, as it has been shown that an increased intake of animal protein could enhance NASH progression. In fact, an ongoing study conducted on more than 3000 patients seems to demonstrate how total protein load could be one of the main factors influencing NASH course and, among them, only animal proteins remained statistically significant after adjusting them by BMI and HOMA index [49].

Moreover, we showed how NASH subject introduce more total daily carbohydrates, and that the intake of soluble ones is higher than starch. This perfectly agrees with scientific evidence: in fact, various studies showed how simple sugars, and especially fructose, could be the basis of metabolic alterations supporting NAFLD: a recent review published in 2015, collecting studies involving both animals and humans, shows how fructose intake could worsen insulin sensitivity, increase de novo lipogenesis and the intrahepatic triglyceride storage, and how a low carb diet could partially reverse those findings [16].

Referring to lipids, i.e. cholesterol, SFA, MUFA and PUFA, we noticed a reduced daily total lipid intake in NASH subjects compared to controls. Despite this, focusing our attention on the type of lipids consumed, we noticed that patients with liver pathology introduce a much higher quantity of SFA and MUFA, and a much lower quantity of PUFA. The role of SFA in NAFLD pathogenesis is well known: first, they have pro-apoptotic effects on different cellular types by inducing stress in the reticulo-endothelial (ER) system, essential for correct intracellular protein folding and for the regulation of Ca^2+^ levels. This could be attributed to a quick assembly of free SFA into saturated phospholipids that are subsequently incorporated in ER membrane, increasing its degree of saturation, which is the critical element for cellular survival and functionality [50]. Furthermore, the increased SFA concentration could be responsible for enhanced mitochondrial activity, caused by an altered intrahepatic triglyceride storage, free fatty acids (FFA) deliver and Krebs cycle hyperfunction, and this could lead to an altered reactive oxygen species (ROS) production and oxidative stress, which are key factors for NAFLD progression [50]. Similarly, the protective role of PUFA is well known, especially omega-3 in the case of metabolic syndrome [51]. Specifically, *n-*3 PUFA, such as alphalinoleic acid and its metabolites, eicosapentaenoic acid (EPA) and docosahexaenoic acid (DHA), demonstrated their action on individual lipidomic profile by reducing tumor necrosis factor (TNF) alpha, interleukin-6 (IL-6), ROS, triglycerides, low-density lipoprotein (LDL) and total serum cholesterol; and by increasing high-density lipoprotein (HDL) levels [52]. A 2012 meta-analysis by Parker et al. collecting the results of nine eligible studies, involving 355 individuals, confirmed a beneficial effect of an *n-*3 PUFA supplementation on the degree of steatosis and on biochemical liver damage markers [51,52,53]. Finally, MUFA showed an important role in preventing NAFLD development: in fact, it has been demonstrated that they could reduce the oxidation of low-density lipoprotein (LDL), serum concentration of LDL and total cholesterol (TC) and triacylglycerols, while decreasing body fat accumulation and postprandial adiponectin expression [52,53,54]. The increased intake observed in our study could simply be attributed to a higher consumption of these fatty acids in our NASH patients, as MUFA is largely comprised in olive oil and in other vegetable oils, thus is abundantly consumed in Italy in the “Mediterranean diet” [55].

Regarding micronutrients, we found some statistically significant alteration in both oligo-elements and vitamins:

Folic acid: The role of folic acid in alcoholic liver disease is already well known, while being less clear in NAFLD. We noticed a statistically relevant increase in the consumption of folic acid in our NASH patients; despite various studies showing a deficiency of this vitamin in NAFLD subjects [56,57], previous studies have not found out any correlation between folic acid serum levels and NAFLD evolution [55,56,57]. Moreover, a study conducted on 10 NASH patients receiving folic acid supplement for six months resulted in no improvements in liver damage markers [58].

Riboflavin: Riboflavin or vitamin B2 has an essential role in redox reaction of numerous metabolic pathways, as well as in cellular respiration, being a fundamental component of flavin adenine dinucleotide (FAD) and flavin mononucleotide (FMN) coenzymes. Nowadays, no studies have correlated this vitamin with NAFLD pathogenesis. The roles of riboflavin, both in immune system modulation [59] and on gut microbiota regulation [60], have been shown as key factors for the progression of metabolic liver disease. Based on this, the reduced intake of vitamin B2 seen in our study could have a critical importance in maintaining this pathology. Further studies are necessary to highlight this aspect more.

Thiamine: Thiamine is poorly stored in human bodies. The importance of a deficiency of this vitamin is well known in alcoholic liver disease (ALD) because of its strongly inhibited absorption caused by intestinal alcohol concentration, which is responsible for diseases such as beri-beri and Korsakoff paralysis. Its role in NAFLD is still poorly known: only one study has shed light on a possible involvement of thiamine in steatosis occurrence. Apparently, it could stimulate a transporter, Organic Cation Transporter-1 (OCT-1), also involved in metformin transport. It has been discovered that knock-out mice for OCT-1 gene expression not only reduced intrahepatic triglycerides storage, possibly using a adenosine monophosphate-activated protein kinase (AMPK) mediated pathway, but also reduced thiamine liver concentration. However, a one-week thiamine-free diet did not show significant effects on body mass and on liver dry weight [61]. Further studies are definitely necessary to better investigate this aspect.

We found a statistically significant increase of vitamin A intake in NASH subjects. It could be explained by the fact that this vitamin is contained many vegetables, but also in butter and cheese, foods usually consumed in higher quantities by NAFLD subject [62].

Even though a beneficial effect of vitamin A on body weight, liver triglycerides and glycogen and degree of steatosis has already been studied, a study demonstrated that vitamin A oral supplements in young mice before they were put on high fat diet (HFD), could lead to a major adiposity, by influencing white adipose tissue proliferative capacity [63].

We noticed a small but statistically valid lower consumption on B6 in NAFLD compared to controls. A 2016 study by Liu et al. showed that in knock-out mice for ApoE, this vitamin could reduce endothelial dysfunction, insulin resistance and triglycerides storage [64]. This could be explained by the action that this vitamin has on the disposal of homocystein and on PUFA metabolism, the latter found reduced in B6 deficient patients.

Vitamin C: its role in NAFLD is still unclear: a study conducted by Oliveira et al. showed that vitamin C could reduce oxidative stress and prevent steatosis development induced by choline deficiency diet-feed (CHD) mice in addition to vitamin E [63,64,65]. Thus far, no study has demonstrated the therapeutic role of vitamin C alone, but only in co-somministration with other well-known antioxidant like vitamin E. Furthermore, even though in a 2016 study of Wei et al. on 3471 patients an inverse relationship between vitamin C intake and the possibility of developing NAFLD was noticed [66], serum levels of this vitamin are not reliable enough to indicate the quantity of vitamin C in oral intake, and even patients that consume recommended quantities of this substance could equally be at risk of having suboptimal serum vitamin C levels [67].

To fully understand the importance of dietary habits in metabolic liver disease pathology, we decided to compare alimentary intakes of NASH patients versus HCV-related liver disease patients. Few studies recorded explored the nutritional sphere of HCV patients. In our study, we noticed that the results found in NAFLD patients regarding their nutritional choices and the consequent considerations that have already been discussed could also be valid for viral liver disease. However, as shown in Table 2 and Table 3, remarkable differences could be noticed regarding carbohydrate and calcium intake as well as unbalance in lipid proportions; a higher intake of calcium could be caused by a greater focus from the healthcare workers in recommending an adequate intake of calcium to prevent bone fractures, because of the well-known role of HCV on bone metabolism and on its capacity in osteoporosis induction [68,69].

Conversely, we found a lower carbohydrate intake and a larger PUFA and MUFA intake, attributed to a greater awareness of infected patients about the importance of their pathology, thus they would be fully adherent of medical prescription, including those regarding the nutrition field. Moreover, NAFLD, being part of metabolic syndrome, needs interventions aimed to modify a unhealthy lifestyle, characterized by sedentary, poor diet and insufficient physical exercise, which are hard correct and maintain [70].

Based on our findings, we were interested to know if patient gender could be a factor impacting nutrition style. To study this, we matched NASH and HCV patients, split by gender, to controls; our results did not find relevant differences in data already discussed in Table 4, regarding an increased caloric intake (only statistically valid for NASH woman, concordantly with a higher number of obese patients compared to males); a reduced lipid intake (only significant for males) with unbalanced SFA/PUFA ratio, a higher intake of carbohydrates and a reduced intake of fiber. With respect to micronutrients, we found interesting data regarding calcium and vitamin D statistically relevant intake in NASH patients. For HCV, it has been emerging that NASH could be an independent factor of poor mineral bone density [71]. Moreover, calcium seems to have a role in NASH pathogenesis and development: a 2016 research by Aslam et al. showed that, in HFD diet-feed mice with calcium supplementation for 18 months, compared to only HFD diet mice, the first one had low inflammation and fibrosis, together with a reduced NAS score at histology. That was explained by its action on gut microbiota, that in calcium supplemented mice appeared to be colonized especially by Ruminococcaceae and Akkermansia, known to be associated with the so-called “healthy gut” for degrading polyglucans and for their action on lipid metabolism, but also by reducing total biliary acids pool (especially taurine-conjugated β-muricholic acid (MCA) and main murine biliary acid) that demonstrated to have citotossic capacity in NASH when present in high concentrations [72].

Vitamin D: The role of vitamin D in NAFLD pathogenesis has been widely discussed; in fact, its healthy effects on glycemic regulation, adiponectin levels, ROS production and intestinal microbiome balance has been already elaborated by various studies, especially by searching for a correlation between serum levels of this vitamin and the onset of metabolic liver pathology [73]. The impact of oral consumption of this nutrient is less clear; even though some trials found a positive effect of vitamin D oral intake on markers of lipid peroxidation and on acute phase protein levels [74], research investigating the effects of high dose vitamin D supplementation in NAFLD subjects versus placebo did not show any improvement on steatosis degree or on metabolic and cardiovascular parameters [75], and same results were seen in another meta-analysis by George et al. about glycemic levels and insulin resistance [76].

Other data concerning zinc intake, previously discussed in our study, showed significantly reduced levels in NASH women. Zinc is a biologically relevant oligo-element for the correct function of proteins involved in various biological processes such as glycemic homeostasis, cell growth and sexual hormones regulation. Many studies showed the protective role of zinc toward alcohol induced liver damage, not only by reducing oxidative stress, but also for its action on intestinal permeability [77]. In the literature, it has already been reported how NASH subjects tend to introduce lower quantity of this oligo-element [29]. A recent study showed how a zinc supplementation, alone or in co-administration with selenium, could improve cholesterol, triglycerides and glucose blood levels, hypothesizing a role in the treatment of this pathology [78].

Finally, we compared patients of the same gender of both pathologies to better understand nutritional differences between two types of liver disease and the impact of gender on those pathologies. Concerning male patients, despite a reduced caloric intake in NASH patients (not statistically significant), we found similar findings that have already been discussed. Similarly, in female patients, outcomes regarding higher caloric and macronutrient intake, with reduced consumption of fiber and of various vitamins except for vitamin D are even clearer. Consequently, although HCV patients still showed poor nutritional habits compared to healthy controls, this table demonstrated that the most evident differences belong to NASH patients, highlighting how the role of alimentation and nutritional choices are very important in the onset and the development of metabolic disease.

Our study had some limitations. Even though we tried to minimize the risk of underreporting as much as possible by carefully instructing the patients in the compilation of the diary, it is not possible to completely avoid this error, with the risk of some under (or even over) estimation of specific nutritional intakes in the collecting of the data. Another possible limitation could be the missing of a direct and pathological correlation between the nutritional assessment in our study and the degree of liver disease evaluated with biopsy samples, making it difficult to link the pathophysiological role of nutrition intakes on the evolution of the pathology; however, we aim to further explore this aspect in a future study.

## 6. Conclusions

In conclusion, this study showed how NASH patient diet, in both males and females, is characterized by profound alteration in macro- and micronutrients composition, and this could be one of the key factors involved in maintaining the metabolic homeostasis necessary for the development of this pathology.

## Figures and Tables

**Table 1:** Main demographic characteristics of controls and liver patient groups.

	Controls		NASH		HCV Chronic Hepatitis	
	M	F	M	F	M	F
**Total Number**	1000	1326	74	50	86	76
**Median Age in Years (Range)**	43 (18–89)	45 (18–89)	47 (39–75)	43 (37–78)	48 (39–66)	51 (44–71)
**BMI**	24.5 ± 1.4	23.6 ± 4.7	31.1 ± 3.7 *	32.7 ± 5.9 *	28.1 ± 6	27 ± 3.5
**Overweight (%)**	17	18	16	19	21	19
**Obese (%)**	0	0	15	18	12	6
**HOMA**	1.7 ± 0.6	1.2 ± 1	3.8 ± 2.2 *	4.1 ± 2 *	2 ± 1.2	1.9 ± 0.9
**AST (IU/L)**	28 ± 11	25 ± 20	96 ± 41 *	77 ± 45 *	51 ± 29	64 ± 30
**ALT (IU/L)**	29 ± 14	37 ± 16	88 ± 65 *	91 ± 61 *	58 ± 15	51 ± 21
**GGT (IU/L)**	35 ± 21	30 ± 13	90 ± 41 *	84 ± 33 *	41 ± 19	38 ± 22

(Age: median; Males = M versus Females = F; Mean ± Standard Deviation). To compare the percent obese and overweight, we performed the Chi-squared test; * *p* < 0.05 vs. Controls. BMI = body mass index; HOMA = homeostatic model assessment; AST = aspartate aminotransferase; ALT = alanine aminotransferase; GGT = gammaglutamyltranspeptidase. IU: international units.

**Table 2:** Intake of macronutrients in study population (Mean ± SD).

	Recommended (LARN)	Controls (*n*: 2326)	NASH (*n*: 124)	*p* vs. Controls	HCV Chronic Hepatitis (*n*: 162)	*p* vs. Controls
*Daily Intake*						
**Total calories** (kcal/day)	M: 2000–2400	2116.5 ± 679.5	2212.3 ± 508.8	0.0235	2082.8 ± 782.9	0.2970
	F: 1800–2300					
**Total proteins** (g/day)	75	80 ± 26	89.7 ± 18.1	**<0.0001**	82.4 ± 28.8	0.1518
**Total proteins** (% of total energy)	15	23 ± 11.5	16.6 ± 3.7	**<0.0001**	16.1 ± 2.1	**<0.0001**
**Animal proteins** (% of total proteins)	40	57.5 ± 24	59.8 ± 12.5	0.0314	60.9 ± 8.3	**<0.0001**
**Vegetal proteins** (%of total proteins)	60	38.5 ± 22.5	40.2 ± 12.5	0.0829	39.1 ± 8.3	0.2274
**Total lipids** (g/day)	65	82.5 ± 29	72.3 ± 17.8	<0.0001	80.5 ± 32.1	0.2207
**Total lipids** (% of total )	30	38.5 ± 19.5	29.8 ± 5.8	**<0.0001**	35.3 ± 6.5	**<0.0001**
**Total carbohydrates** (g/day)	290	292.5 ± 100	317.5 ± 110.1	0.0073	267.6 ± 117.7	0.0047
**Total carbohydrates** (% of total energy)	55	57 ± 10	56.6 ± 7.7	0.2902	50.82 ± 7.03	**<0.0001**
**Amide** (g/day)	220	190 ± 90	200.1 ± 93.5	0.1212	175.78 ± 88.13	0.0244
**Soluble carbohydrates** (g/day)	70	89.5 ± 26.5	99.6 ± 23.7	**<0.0001**	79.8 ± 29.2	**<0.0001**
**Fiber** (g/day)	23	27.5 ± 10	27.8 ± 10.9	0.3824	25.9 ± 7.7	0.0066
**Cholesterol** (mg/day)	255	238.5 ± 100	253.5 ± 99.5	0.0521	244.1 ± 102	0.2457
**Saturated fatty acids** (% of total )	7	8 ± 3.95	10.4 ± 2.9	**<0.0001**	12.5 ± 2.5	**<0.0001**
**Polyunsaturated fatty acids** (% of total)	18	22.5 ± 6.5	5.5 ± 2.3	**<0.0001**	6.5 ± 2.1	**<0.0001**
**Monounsaturated fatty acids** (% of total)	4	4.55 ± 1.3	12.5 ± 3.4	**<0.0001**	15.1 ± 4.8	**0.0001**

LARN: Nutrition and Energy Reference Assuming Levels; NASH: non-alcoholic steatohepatitis; HCV: hepatitis C virus patients. Two samples *t*-test comparison of means and standard deviations, unequal variances assumed, with Welch approximation. The Bonferroni multiple comparison post-hoc correction (α/n variables) for the above tests is 0.05/16 = 0.003125. The significances were highlighted accordingly.

**Table 3:** Intake of micronutrients in study population (Mean ± SD).

	Recommended (LARN)	Controls (*n*: 2326)	NASH (*n*: 124)	*p* vs. Controls	HCV Chronic Hepatitis (*n*: 162)	*p* vs. Controls
*Daily Intake*						
**Calcium** (mg)	M: 1200; F: 1500	816.5 ± 314	833.1 ± 328.2	0.2916	910.5 ± 261.4	**<0.0001**
**Iron** (mg)	18	12.5 ± 3.5	13.1 ± 2.9	0.0140	12.3 ± 3.5	0.2414
**Zinc** (mg)	7	11.23 ± 3.5	10.4 ± 4.1	0.0143	10.6 ± 3.1	0.0070
**Folic acid** (µg)	200	342 ± 101.5	400.2 ± 153.9	**<0.0001**	367.1 ± 104.2	**0.0017**
**Niacin** (mg)	14	14.5 ± 4.5	14.9 ± 3.5	0.1122	14.2 ± 5.4	0.2454
**Riboflavin** (mg)	1.2	3.5 ± 2.5	2.8 ± 1.6	**<0.0001**	3.9 ± 3.7	0.0887
**Thiamine** (mg)	0.9	0.9 ± 0.4	1 ± 0.3	**0.0003**	1 ± 0.4	**0.0012**
**Vitamin A** (µg)	600	723 ± 199.5	931.6 ± 352.7	**<0.0001**	859.4 ± 264.2	**<0.0001**
**Vitamin B6** (mg)	1.1	2 ± 0.5	1.8 ± 0.3	**<0.0001**	1.72 ± 0.52	**<0.0001**
**Vitamin C** (mg)	70	118 ± 50.5	150.7 ± 55	**<0.0001**	140.8 ± 47.5	**<0.0001**
**Vitamin D** (µg)	10	1.8 ± 0.9	1.84 ± 1.83	0.4046	1.6 ± 1	0.0071
**Vitamin E** (mg)	8	6.5 ± 2	7.44 ± 3.98	0.0050	6.6 ± 1.6	0.2254

LARN: Nutrition and Energy Reference Assuming Levels; NASH: non-alcoholic steatohepatitis; HCV: hepatitis C virus patients. Two samples *t*-test comparison of means and standard deviations, unequal variances assumed, with Welch approximation. The Bonferroni multiple comparison post-hoc correction (α/n variable) for the above tests is 0.05/12 = 0.004167. The significances were highlighted accordingly.

**Table 4:** Intake of macronutrients in study population (↓↑ indicate variations in respect to amounts recommended in Italy) (Males = M versus Females = F; Mean ± Standard Deviation).

	Recommended (LARN)	Controls		NASH		*p* NASH vs. Control		HCV Chronic Hepatitis		*p* HCV vs. Control	
*Daily Intake*		M (*n*: 1000)	F (*n*: 1326)	M (*n*: 74)	F (*n*: 50)	M	F	M (*n*: 86)	F (*n*: 76)	M	F
**Total calories** (kcal)	M: 2000–2400; F: 1800–2300	2361 ± 969	1872 ± 390	2208.5 ± 560.8	2230.9 ± 129.1	0.0180	**<0.0001**	2350.5 ± 1010.8	1831.9 ± 348.3	0.4631	0.1622
**Total proteins** (g)	75	81 ± 36 ↑	79 ± 16 ↑	91.3 ± 19.5 ↑	81.3 ± 5.1 ↑	0.2160	0.0039	89.5 ± 38.2 ↑	75.2 ± 13.7	0.0247	0.0111
**Total proteins** (% of total energy)	15	24 ± 12 ↑	22 ± 11 ↑	17 ± 3.9	14.5 ± 0.1	**<0.0001**	**<0.0001**	15.4 ± 1.8	16.5 ± 2.1	**<0.0001**	**<0.0001**
**Animal proteins** (% of total proteins)	40	61 ± 26 ↑	54 ± 22 ↑	59.9 ± 13.3 ↑	59.4 ± 11.1 ↑	0.2655	**0.0010**	59.4 ± 9.7 ↑	62.3 ± 6.7 ↑	0.1152	**<0.0001**
**Vegetal proteins** (%of total proteins)	60	37 ± 18 ↓	40 ± 27 ↓	40.1 ± 13.3 ↓	40.5 ± 11.1 ↓	0.0315	0.3871	40.6 ± 9.7 ↓	37.6 ± 6.7 ↓	0.0015	0.0127
**Total lipids** (g)	65	81 ± 31 ↑	84 ± 27 ↑	70.6 ± 18.8	80.9 ± 11.2 ↑	**<0.0001**	0.0402	89.9 ± 40.3 ↑	72.6 ± 18.8	0.0247	**<0.0001**
**Total lipids** (% of total energy)	30	38 ± 11 ↑	39 ± 28 ↑	29.2 ± 6.2	32.5 ± 2.6	**<0.0001**	**<0.0001**	34.7 ± 6.6	35.7 ± 6.6	**<0.0001**	**0.0012**
**Cholesterol** (mg)	255	235 ± 100	242 ± 100	250.6 ± 104.1	267.7 ± 104.9	0.1079	0.0472	264.2 ± 127.5	222.4 ± 68	0.0206	0.0099
**Saturated fatty acids** (g)	4.5	8.5 ± 3.9 ↑	7.5 ± 4 ↑	21 ± 12.1 ↑	15.4 ± 3.9 ↑	**<0.0001**	**<0.0001**	12.5 ± 3 ↑	12.4 ± 2.1 ↑	**<0.0001**	**<0.0001**
**Polyunsaturated fatty acids** (g)	13	22 ± 6 ↑	23 ± 7 ↑	10 ± 5.9	6.3 ± 2.4 ↓	**<0.0001**	**<0.0001**	6.6 ± 2.5 ↓	6.5 ± 1.6 ↓	**<0.0001**	**<0.0001**
**Monounsaturated fatty acids** (g)	2.6	4.6 ± 0.9	4.5 ± 1.7	35.3 ± 21.3 ↑	39.7 ± 15.8 ↑	**<0.0001**	**<0.0001**	14.4 ± 3.9 ↑	15.7 ± 5.5 ↑	**<0.0001**	**<0.0001**
**Total carbohydrates** (g)	290	298 ± 90	287 ± 110	356.5 ± 115.6 ↑	324.4 ± 87.1 ↑	**<0.0001**	**0.0025**	303.5 ± 152.8	232.4 ± 56.1	0.3715	**<0.0001**
**Total carbohydrates** (% of total energy)	55	55 ± 12	59 ± 8	56.7 ± 8.4	56.1 ± 2.9	0.0539	**<0.0001**	51.1 ± 8.1	50.5 ± 6.1	**<0.0001**	**<0.0001**
**Amide** (g)	220	200 ± 93	180 ± 87	200.5 ± 103.2	198.1 ± 15.9	0.4839	**<0.0001**	202.7 ± 113.8	148.9 ± 43.4	0.4155	**<0.0001**
**Soluble carbohydrates** (g)	70	99 ± 23 ↑	80 ± 30 ↑	159 ± 31 ↑	128 ± 21 ↑	**<0.0001**	**<0.0001**	86.5 ± 34.2 ↑	73.5 ± 22.1	**0.0006**	0.0083
**Fiber** (g)	23	28 ± 12	27 ± 8	15 ± 2.8	14.2 ± 2.8	**<0.0001**	**<0.0001**	26.7 ± 7.6	25.1 ± 7.8	0.0763	**0.0015**

Two samples *t*-test comparison of means and standard deviations, unequal variances assumed, with Welch approximation. The Bonferroni multiple comparison post-hoc correction (α/n) for the above tests is 0.05/16 = 0.003125. The significances were highlighted accordingly.

**Table 5:** Intake of micronutrients in study population (↓↑ indicate variations in respect to amounts recommended in Italy) (Mean ± SD).

	Recommended (LARN)	Controls		NASH		*p* NASH M/F		HCV Chronic Hepatitis		*p* HCV M/F	
*Dailyintake*		M (*n*: 1000)	F (*n*: 1326)	M (*n*: 74)	F (*n*: 50)	M	F	M (*n*: 86)	F (*n*: 76)	M	F
**Calcium** (mg)	M:1200; F:1500	833 ± 328 ↓	800 ± 300 ↓	869.1 ± 350.8 ↓	653.2 ± 6.4 ↓	0.1967	**<0.0001**	944.1 ± 321.3 ↓	873.1 ± 214.3 ↓	0.0014	0.0029
**Iron** (mg)	18	13 ± 3 ↓	12 ± 4 ↓	13.1 ± 3.2 ↓	12.8 ± 0.3 ↓	0.3976	0.0156	13.1 ± 4.1 ↓	11.4 ± 2.9 ↓	0.4127	0.0451
**Zinc** (mg)	7	10.4 ± 3	12.4 ± 4	11.3 ± 2.9	9.7 ± 1.4	0.0060	**<0.0001**	10.9 ± 3.5	10.2 ± 2.1	0.1010	**<0.0001**
**Folic acid** (µg)	200	364 ± 104 ↑	320 ± 99 ↑	406.1 ± 167.1 ↑	370.6 ± 86.9 ↑	0.0179	**0.0001**	346.2 ± 111.4 ↑	381.6 ± 125.3 ↑	0.0758	**<0.0001**
**Niacin** (mg)	14	15 ± 5	14 ± 4	15.6 ± 3.7	13.6 ± 2.1	0.0968	0.1055	14.9 ± 6.4	13.3 ± 3.1	0.4440	0.0316
**Riboflavin** (mg)	1.2	3 ± 2	4 ± 3	2.9 ± 1.8	2.1 ± 0.7	0.3242	**<0.0001**	4.56 ± 4.91	3.28 ± 2.21	0.0022	0.0041
**Thiamine** (mg)	0.9	1 ± 0.4	0.8 ± 0.4	1 ± 0.4	1 ± 0.1	0.5	**<0.0001**	1.1 ± 0.6	1.1 ± 0.4	0.0664	**<0.0001**
**Vitamin A** (µg)	600	664 ± 276	782 ± 123	956.1 ± 383 ↑	808.8 ± 109.3 ↑	**<0.0001**	0.0480	870.4 ± 210.4 ↑	848.2 ± 303.4 ↑	**<0.0001**	0.0311
**Vitamin B6** (mg)	1.1	2 ± 0.4	2 ± 0.6	1.9 ± 0.3	1.6 ± 0.1	0.0042	**<0.0001**	1.7 ± 0.5	1.5 ± 0.5	**<0.0001**	**<0.0001**
**Vitamin C** (mg)	70	106 ± 55 ↑	130 ± 46 ↑	156.2 ± 57.8 ↑	123.2 ± 36.3 ↑	**<0.0001**	0.1019	134.5 ± 38.4 ↑	147 ± 53.6 ↑	**<0.0001**	0.0041
**Vitamin D** (µg)	10	2 ± 0.8 ↓	1.6 ± 1 ↓	1.7 ± 1 ↓	2.1 ± 0.1 ↓	0.0068	**<0.0001**	1.9 ± 1.1 ↓	1.2 ± 0.8 ↓	0.2059	**<0.0001**
**Vitamin E** (mg)	8	7 ± 2	6 ± 2	7.7 ± 4.3	6.1 ± 0.1	0.0844	0.0390	6.6 ± 1.2	6.6 ± 1.8	0.0264	0.0031

Two samples *t*-test comparison of means and standard deviations, unequal variances assumed, with Welch approximation. The Bonferroni multiple comparison post-hoc correction (α/n) for the above tests is 0.05/12 = 0.004167. The significances were highlighted accordingly.

**Table 6:** Intake of macronutrients in study population in males and females (NASH versus HCV-chronic hepatitis (Mean ± SD)).

	Males			Females		
Daily Intake	NASH (*n*: 74)	HCV Chronic Hepatitis (*n*: 86)	*p*	NASH (*n*: 50)	HCV Chronic Hepatitis (*n*: 76)	*p*
**Total calories** (kcal)	2208.57 ± 560.82	2350.5 ± 1010.8	0.1423	2230.9 ±129.1	1831.9 ± 348.3	**<0.0001**
**Total proteins** (g)	91.3 ± 19.5	89.5 ± 38.2	0.3573	81.3 ±5.1	75.2 ± 13.7	**0.0016**
**Total proteins** (% of total energy)	17 ± 3.9	15.4 ± 1.8	**0.0004**	14.5 ±0.1	16.5 ± 2.1	**<0.0001**
**Animal proteins** (% of total proteins)	59.9 ± 13.3	59.4 ± 9.7	0.3922	59.4±11.1	62.3 ± 6.7 ↑	0.0349
**Vegetal proteins** (%of total proteins)	40.1 ± 13.3	40.6 ± 9.7	0.3922	40.5±11.1	37.6 ± 6.7 ↓	0.0349
**Total lipids** (g)	70.6 ± 18.8	89.9 ± 40.3	**0.0001**	80.9±11.2	72.6 ± 18.8	**0.0029**
**Total lipids** (% of total energy)	29.2 ± 6.2	34.7 ± 6.6	**<0.0001**	32.5 ± 2.6	35.7 ± 6.6	**<0.0001**
**Cholesterol** (mg)	250.6 ± 104.1	264.2 ± 127.5	0.2328	267.7 ± 104.9	222.4 ± 68	**0.0019**
**Saturated fatty acids** (g)	21 ± 12.1	12.5 ± 3	**<0.0001**	15.4 ± 3.9	12.4 ± 2.1 ↑	**<0.0001**
**Polyunsaturated fatty acids** (g)	10 ± 5.9	6.6 ± 2.5	**<0.0001**	6.3 ± 2.4	6.5 ± 1.6 ↓	0.2877
**Monounsaturated fatty acids** (g)	35.3 ± 21.3	14.4 ± 3.9	**<0.0001**	39.7 ± 15.8	15.7 ± 5.5 ↑	**<0.0001**
**Total carbohydrates** (g)	356.5 ± 115.6	303.5 ± 152.8	0.0079	324.4 ± 87.1	232.4 ± 56.1	**<0.0001**
**Total carbohydrates** (% of total energy)	56.7 ± 8.4	51.1 ± 8.1	**<0.0001**	56.1 ± 2.9	50.5 ± 6.1	**<0.0001**
**Amide** (g)	200.5 ± 103.2	202.7 ± 113.8	0.4494	198.1 ± 15.9	148.9 ± 43.4	**<0.0001**
**Soluble carbohydrates** (g)	159 ± 31	86.5 ± 34.2	**<0.0001**	128 ± 21	73.5 ± 22.1	**<0.0001**
**Fiber** (g)	15 ± 2.8	26.7 ± 7.6	**<0.0001**	14.2 ± 2.8	25.1 ± 7.8	**<0.0001**

Two samples *t*-test comparison of means and standard deviations, unequal variances assumed, with Welch approximation. The Bonferroni multiple comparison post-hoc correction (α/n) for the above tests is 0.05/16 = 0.003125. The significances were highlighted accordingly.

**Table 7:** Intake of micronutrients in study population in males and females (NASH versus HCV-chronic hepatitis (Mean ± SD)).

	Males			Females		
Daily Intake	NASH (*n*: 74)	HCV Chronic Hepatitis (*n*: 86)	*p*	NASH (*n*: 50)	HCV Chronic Hepatitis (*n*: 76)	*p*
**Calcium** (mg)	869.1 ± 350.8 ↓	944.1 ± 321.3 ↓	0.0801	653.2 ± 6.4 ↓	873.1 ± 214.3 ↓	**<0.0001**
**Iron** (mg)	13.1 ± 3.2 ↓	13.1 ± 4.1 ↓	0.5000	12.8 ± 0.3 ↓	11.4 ± 2.9 ↓	**0.0005**
**Zinc** (mg)	11.3 ± 2.9	10.9 ± 3.5	0.2184	9.7 ± 1.4	10.2 ± 2.1	0.0707
**Folic acid** (µg)	406.1 ± 167.1 ↑	346.2 ± 111.4 ↑	**0.0038**	370.6 ± 86.9 ↑	381.6 ± 125.3 ↑	0.2948
**Niacin** (mg)	15.6 ± 3.7	14.9 ± 6.4	0.2042	13.6 ± 2.1	13.3 ± 3.1	0.2750
**Riboflavin** (mg)	2.9 ± 1.8	4.56 ± 4.91	**0.0033**	2.1 ± 0.7	3.28 ± 2.21	**0.0002**
**Thiamine** (mg)	1 ± 0.4	1.1 ± 0.6	0.1123	1 ± 0.1	1.1 ± 0.4	0.0430
**Vitamin A** (µg)	956.1 ± 383 ↑	870.4 ± 210.4 ↑	0.0380	808.8 ± 109.3 ↑	848.2 ± 303.4 ↑	0.1902
**Vitamin B6** (mg)	1.9 ± 0.3	1.7 ± 0.5	0.0150	1.6 ± 0.1	1.5 ± 0.5	0.0829
**Vitamin C** (mg)	156.2 ± 57.8 ↑	134.5 ± 38.4 ↑	**0.0026**	123.2 ± 36.3 ↑	147 ± 53.6 ↑	**0.0018**
**Vitamin D** (µg)	1.7 ± 1 ↓	1.9 ± 1.1 ↓	0.1168	2.1 ± 0.1 ↓	1.2 ± 0.8 ↓	**<0.0001**
**Vitamin E** (mg)	7.7 ± 4.3	6.6 ± 1.2	0.0122	6.1 ± 0.1	6.6 ± 1.8	0.0091

Two samples *t*-test comparison of means and standard deviations, unequal variances assumed, with Welch approximation. The Bonferroni multiple comparison post-hoc correction (α/n) for the above tests is 0.05/12 = 0.004167. The significances were highlighted accordingly.
